# Supplementary material for: Increasing Resistance and Changes in Distribution of Serotypes of Streptococcus agalactiae in Poland
Source: Pathogens. 2020 Jun 29;9(7):526. doi: 10.3390/pathogens9070526 (PMC7400139; doi:10.3390/pathogens9070526)
Supplement: Supplementary file 1 [file pathogens-09-00526-s001.pdf]

Supplementary Table S1. Antimicrobial susceptibility testing results obtained by disc diffusion method and antimicrobial gradient diffusion method (E-test).

| | E-test | Disc diffusion method | | | | | | | | | | | | Resistance mechanisms |
|---|---|---|---|---|---|---|---|---|---|---|---|---|---|---|
| | Penicilin G | P * (1IU) | | TE* (30µg) | | TGC* (15 µg) | | LEV* (5 µg) | | E* (15 µg) | | DA* (2 µg) | | |
| Strains derived from pregnant women (n = 85) | | | | | | | | | | | | | | |
| 1 | 0.094 | 22 | S | 10 | R | 20 | S | 19 | S | 26 | S | 23 | S | - |
| 3 | 0.094 | 22 | S | 8 | R | 19 | S | 19 | S | 6 | R | 6 | R | $MLS_B$ c |
| 4 | 0.064 | 25 | S | 8 | R | 20 | S | 20 | S | 27 | S | 25 | S | - |
| 7 | 0.094 | 21 | S | 9 | R | 19 | S | 19 | S | 25 | S | 22 | S | - |
| 8 | 0.094 | 22 | S | 13 | R | 21 | S | 23 | S | 24 | S | 22 | S | - |
| 9 | 0.094 | 23 | S | 8 | R | 19 | S | 20 | S | 25 | S | 22 | S | - |
| 10 | 0.064 | 22 | S | 11 | R | 22 | S | 23 | S | 28 | S | 22 | S | - |
| 11 | 0.125 | 23 | S | 8 | R | 20 | S | 20 | S | 27 | S | 24 | S | - |
| 12 | 0.064 | 23 | S | 14 | R | 19 | S | 20 | S | 24 | S | 21 | S | - |
| 13 | 0.094 | 24 | S | 8 | R | 19 | S | 20 | S | 12 | R | 19 | R | $MLS_B$ i |
| 14 | 0.064 | 23 | S | 8 | R | 20 | S | 19 | S | 6 | R | 6 | R | $MLS_B$ c |
| 17 | 0.094 | 21 | S | 8 | R | 21 | S | 20 | S | 25 | S | 22 | S | - |
| 19 | 0.064 | 22 | S | 8 | R | 19 | S | 19 | S | 25 | S | 23 | S | - |
| 20 | 0.094 | 23 | S | 12 | R | 21 | S | 21 | S | 28 | S | 24 | S | - |
| 21 | 0.064 | 25 | S | 26 | S | 21 | S | 22 | S | 26 | S | 22 | S | - |
| 22 | 0.094 | 23 | S | 8 | R | 19 | S | 19 | S | 13 | R | 12 | R | $MLS_B$ c |
| 23 | 0.032 | 25 | S | 8 | R | 21 | S | 24 | S | 28 | S | 25 | S | - |
| 24 | 0.064 | 23 | S | 10 | R | 22 | S | 20 | S | 26 | S | 25 | S | - |
| 27 | 0.094 | 24 | S | 8 | R | 19 | S | 19 | S | 6 | R | 6 | R | $MLS_B$ c |
| 30 | 0.125 | 25 | S | 15 | R | 21 | S | 24 | S | 24 | S | 22 | S | - |
| 31 | 0.094 | 23 | S | 8 | R | 19 | S | 20 | S | 25 | S | 22 | S | - |
| 35 | 0.064 | 22 | S | 10 | R | 20 | S | 20 | S | 25 | S | 22 | S | - |
| 36 | 0.094 | 24 | S | 15 | R | 21 | S | 20 | S | 6 | R | 6 | R | $MLS_B$ c |
| 40 | 0.094 | 23 | S | 10 | R | 19 | S | 21 | S | 25 | S | 22 | S | - |
| 41 | 0.064 | 24 | S | 12 | R | 21 | S | 20 | S | 26 | S | 23 | S | - |
| 42 | 0.094 | 24 | S | 13 | R | 20 | S | 20 | S | 26 | S | 22 | S | - |
| 43 | 0.094 | 22 | S | 10 | R | 19 | S | 19 | S | 24 | S | 21 | S | - |
| 44 | 0.064 | 22 | S | 8 | R | 20 | S | 19 | S | 25 | S | 21 | S | - |
| 45 | 0.094 | 22 | S | 8 | R | 19 | S | 19 | S | 23 | S | 22 | S | - |
| 46 | 0.032 | 26 | S | 23 | S | 21 | S | 20 | S | 27 | S | 24 | S | - |
| 49 | 0.064 | 23 | S | 10 | R | 22 | S | 20 | S | 12 | R | 6 | R | $MLS_B$ c |
| 51 | 0.094 | 21 | S | 10 | R | 19 | S | 20 | S | 25 | S | 22 | S | - |
| 53 | 0.094 | 22 | S | 12 | R | 20 | S | 19 | S | 25 | S | 23 | S | - |
| 54 | 0.064 | 23 | S | 10 | R | 19 | S | 20 | S | 6 | R | 6 | R | $MLS_B$ c |
| 57 | 0.047 | 25 | S | 11 | R | 19 | S | 18 | S | 25 | S | 21 | S | - |
| 59 | 0.094 | 23 | S | 9 | R | 19 | S | 20 | S | 23 | S | 21 | S | - |
| 60 | 0.094 | 22 | S | 10 | R | 20 | S | 19 | S | 26 | S | 23 | S | - |
| 61 | 0.094 | 23 | S | 17 | R | 24 | S | 24 | S | 8 | R | 19 | R | $MLS_B$ i |

| | | | | | | | | | | | | | | |
|---|---|---|---|---|---|---|---|---|---|---|---|---|---|---|
| 62 | 0.047 | 22 | S | 20 | R | 19 | S | 20 | S | 25 | S | 22 | S | - |
| 64 | 0.125 | 21 | S | 12 | R | 21 | S | 21 | S | 25 | S | 22 | S | - |
| 65 | 0.094 | 21 | S | 12 | R | 19 | S | 20 | S | 25 | S | 22 | S | - |
| 66 | 0.125 | 22 | S | 13 | R | 19 | S | 21 | S | 25 | S | 23 | S | - |
| 67 | 0.064 | 25 | S | 10 | R | 20 | S | 19 | S | 26 | S | 22 | S | - |
| 68 | 0.094 | 23 | S | 18 | R | 24 | S | 22 | S | 30 | S | 25 | S | - |
| 69 | 0.094 | 26 | S | 14 | R | 20 | S | 22 | S | 28 | S | 24 | S | - |
| 70 | 0.094 | 23 | S | 8 | R | 20 | S | 20 | S | 26 | S | 23 | S | - |
| 71 | 0.125 | 22 | S | 10 | R | 19 | S | 20 | S | 26 | S | 23 | S | - |
| 72 | 0.047 | 22 | S | 8 | R | 19 | S | 20 | S | 17 | R | 20 | R | $MLS_B$ i |
| 75 | 0.064 | 26 | S | 26 | S | 22 | S | 23 | S | 27 | S | 22 | S | - |
| 78 | 0.064 | 30 | S | 12 | R | 21 | S | 24 | S | 6 | R | 6 | R | $MLS_B$ c |
| 80 | 0.094 | 24 | S | 10 | R | 19 | S | 19 | S | 6 | R | 6 | R | $MLS_B$ c |
| 81 | 0.047 | 22 | S | 10 | R | 19 | S | 20 | S | 26 | S | 23 | S | - |
| 82 | 0.064 | 25 | S | 10 | R | 22 | S | 22 | S | 28 | S | 26 | S | - |
| 83 | 0.094 | 23 | S | 9 | R | 19 | S | 19 | S | 25 | S | 21 | S | - |
| 86 | 0.125 | 22 | S | 17 | R | 21 | S | 19 | S | 24 | S | 21 | S | - |
| 88 | 0.094 | 18 | S | 12 | R | 20 | S | 22 | S | 26 | S | 23 | S | - |
| 90 | 0.094 | 23 | S | 10 | R | 19 | S | 21 | S | 25 | S | 22 | S | - |
| 91 | 0.094 | 22 | S | 20 | R | 19 | S | 20 | S | 25 | S | 22 | S | - |
| 94 | 0.064 | 23 | S | 10 | R | 19 | S | 20 | S | 26 | S | 23 | S | - |
| 96 | 0.064 | 24 | S | 10 | R | 19 | S | 21 | S | 14 | R | 6 | R | $MLS_B$ c |
| 98 | 0.047 | 27 | S | 14 | R | 21 | S | 24 | S | 27 | S | 24 | S | - |
| 99 | 0.094 | 25 | S | 29 | S | 20 | S | 21 | S | 28 | S | 19 | S | - |
| 106 | 0.094 | 26 | S | 13 | R | 20 | S | 21 | S | 26 | S | 23 | S | - |
| 107 | 0.064 | 26 | S | 14 | R | 21 | S | 21 | S | 10 | R | 23 | S | M |
| 111 | 0.064 | 26 | S | 15 | R | 22 | S | 24 | S | 25 | S | 24 | S | - |
| 112 | 0.064 | 22 | S | 14 | R | 21 | S | 24 | S | 16 | R | 19 | R | $MLS_B$ i |
| 113 | 0.064 | 28 | S | 14 | R | 22 | S | 24 | S | 29 | S | 24 | S | - |
| 114 | 0.094 | 23 | S | 14 | R | 20 | S | 21 | S | 25 | S | 22 | S | - |
| 115 | 0.064 | 25 | S | 25 | S | 21 | S | 22 | S | 25 | S | 22 | S | - |
| 117 | 0.064 | 25 | S | 18 | R | 22 | S | 25 | S | 6 | R | 6 | R | $MLS_B$ c |
| 118 | 0.094 | 27 | S | 23 | S | 22 | S | 24 | S | 27 | S | 24 | S | - |
| 119 | 0.094 | 24 | S | 14 | R | 21 | S | 21 | S | 28 | S | 25 | S | - |
| 121 | 0.125 | 23 | S | 12 | R | 20 | S | 20 | S | 25 | S | 23 | S | - |
| 122 | 0.094 | 27 | S | 15 | R | 24 | S | 25 | S | 14 | R | 21 | R | $MLS_B$ i |
| 123 | 0.064 | 28 | S | 14 | R | 21 | S | 22 | S | 15 | R | 22 | R | $MLS_B$ i |
| 124 | 0.032 | 26 | S | 13 | R | 22 | S | 25 | S | 6 | R | 6 | R | $MLS_B$ c |
| 125 | 0.094 | 25 | S | 14 | R | 21 | S | 22 | S | 20 | S | 23 | S | - |
| 127 | 0.064 | 30 | S | 17 | R | 22 | S | 21 | S | 6 | R | 6 | R | $MLS_B$ c |
| 128 | 0.094 | 25 | S | 18 | R | 20 | S | 22 | S | 25 | S | 17 | S | - |
| 129 | 0.094 | 25 | S | 28 | S | 22 | S | 22 | S | 28 | S | 24 | S | - |
| 130 | 0.064 | 27 | S | 12 | R | 21 | S | 23 | S | 28 | S | 25 | S | - |
| 131 | 0.064 | 26 | S | 13 | R | 22 | S | 22 | S | 28 | S | 24 | S | - |
| 138 | 0.047 | 28 | S | 16 | R | 20 | S | 24 | S | 28 | S | 25 | S | - |

| | | | | | | | | | | | | | | |
|---|---|---|---|---|---|---|---|---|---|---|---|---|---|---|
| 141 | 0.094 | 28 | S | 18 | R | 23 | S | 25 | S | 17 | R | 25 | S | M |
| 146 | 0.094 | 25 | S | 11 | R | 19 | S | 22 | S | 6 | R | 6 | R | $MLS_B$ c |
| Strains derived from other adults (n = 44) | | | | | | | | | | | | | | |
| 50 | 0.094 | 25 | S | 14 | R | 20 | S | 26 | S | 27 | S | 25 | S | - |
| 63 | 0.064 | 27 | S | 19 | R | 22 | S | 24 | S | 21 | S | 19 | S | - |
| 76 | 0.032 | 28 | S | 17 | R | 23 | S | 25 | S | 28 | S | 27 | S | - |
| 93 | 0.064 | 26 | S | 13 | R | 22 | S | 19 | S | 27 | S | 25 | S | - |
| 97 | 0.064 | 27 | S | 16 | R | 22 | S | 23 | S | 30 | S | 25 | S | - |
| 100 | 0.094 | 25 | S | 13 | R | 22 | S | 23 | S | 15 | R | 22 | R | $MLS_B$ i |
| 101 | 0.094 | 25 | S | 11 | R | 21 | S | 23 | S | 26 | S | 25 | S | - |
| 105 | 0.047 | 31 | S | 17 | R | 23 | S | 22 | S | 28 | S | 24 | S | - |
| 116 | 0.094 | 25 | S | 15 | R | 21 | S | 24 | S | 25 | S | 23 | S | - |
| 142 | 0.064 | 26 | S | 25 | S | 21 | S | 23 | S | 26 | S | 24 | S | - |
| 148 | 0.125 | 23 | S | 13 | R | 22 | S | 20 | S | 24 | S | 22 | S | - |
| 149 | 0.047 | 27 | S | 17 | R | 20 | S | 20 | S | 27 | S | 22 | S | - |
| 153 | 0.125 | 23 | S | 17 | R | 20 | S | 21 | S | 25 | S | 24 | S | - |
| 154 | 0.064 | 25 | S | 15 | R | 22 | S | 22 | S | 27 | S | 24 | S | - |
| 157 | 0.047 | 24 | S | 13 | R | 20 | S | 21 | S | 14 | R | 21 | R | $MLS_B$ i |
| 159 | 0.047 | 28 | S | 12 | R | 22 | S | 24 | S | 6 | R | 6 | R | $MLS_B$ c |
| 163 | 0.094 | 28 | S | 13 | R | 19 | S | 21 | S | 6 | R | 6 | R | $MLS_B$ c |
| 165 | 0.064 | 26 | S | 26 | S | 24 | S | 22 | S | 28 | S | 25 | S | - |
| 168 | 0.094 | 25 | S | 11 | R | 20 | S | 21 | S | 14 | R | 13 | R | $MLS_B$ c |
| 170 | 0,064 | 24 | S | 12 | R | 21 | S | 21 | S | 6 | R | 6 | R | $MLS_B$ c |
| 171 | 0.064 | 26 | S | 14 | R | 21 | S | 24 | S | 26 | S | 23 | S | - |
| 172 | 0.064 | 27 | S | 13 | R | 27 | S | 24 | S | 6 | R | 6 | R | $MLS_B$ c |
| 173 | 0.094 | 26 | S | 14 | R | 21 | S | 22 | S | 28 | S | 25 | S | - |
| 174 | 0.094 | 24 | S | 14 | R | 21 | S | 25 | S | 27 | S | 24 | S | - |
| 175 | 0.064 | 26 | S | 12 | R | 21 | S | 23 | S | 6 | R | 6 | R | $MLS_B$ c |
| 177 | 0.094 | 26 | S | 13 | R | 22 | S | 24 | S | 29 | S | 26 | S | - |
| 179 | 0.064 | 26 | S | 14 | R | 19 | S | 23 | S | 14 | R | 21 | R | $MLS_B$ i |
| 180 | 0.094 | 25 | S | 17 | R | 23 | S | 22 | S | 28 | S | 24 | S | - |
| 181 | 0.064 | 25 | S | 15 | R | 21 | S | 24 | S | 26 | S | 24 | S | - |
| 182 | 0.047 | 29 | S | 16 | R | 20 | S | 23 | S | 24 | S | 22 | S | - |
| 183 | 0.094 | 26 | S | 17 | R | 22 | S | 24 | S | 17 | R | 23 | S | M |
| 184 | 0.032 | 26 | S | 16 | R | 25 | S | 23 | S | 26 | S | 23 | S | - |
| 185 | 0.064 | 26 | S | 17 | R | 24 | S | 23 | S | 29 | S | 24 | S | - |
| 186 | 0.047 | 26 | S | 13 | R | 21 | S | 21 | S | 26 | S | 24 | S | - |
| 188 | 0.064 | 27 | S | 16 | R | 21 | S | 22 | S | 28 | S | 25 | S | - |
| 189 | 0.094 | 25 | S | 17 | R | 21 | S | 23 | S | 28 | S | 24 | S | - |
| 18 | 0.064 | 27 | S | 25 | S | 22 | S | 21 | S | 28 | S | 23 | S | - |
| 58 | 0.125 | 24 | S | 18 | R | 21 | S | 22 | S | 16 | R | 6 | R | $MLS_B$ c |
| 77 | 0.064 | 26 | S | 12 | R | 21 | S | 22 | S | 6 | R | 6 | R | $MLS_B$ c |
| 133 | 0.047 | 29 | S | 16 | R | 24 | S | 23 | S | 29 | S | 25 | S | - |
| 143 | 0.064 | 26 | S | 17 | R | 27 | S | 24 | S | 16 | R | 26 | S | M |
| 147 | 0.064 | 25 | S | 14 | R | 21 | S | 23 | W | 14 | R | 21 | R | $MLS_B$ i |

| | | | | | | | | | | | | | | |
|---|---|---|---|---|---|---|---|---|---|---|---|---|---|---|
| 161 | 0.064 | 27 | S | 19 | R | 22 | S | 26 | S | 31 | S | 25 | S | - |
| 164 | 0.094 | 25 | S | 15 | R | 22 | S | 26 | S | 26 | S | 24 | S | - |
| Strains derived from newborn (n = 36) | | | | | | | | | | | | | | |
| 2 | 0.094 | 25 | S | 14 | R | 21 | S | 20 | S | 26 | S | 22 | S | - |
| 5 | 0.064 | 25 | S | 13 | R | 22 | S | 23 | S | 17 | R | 21 | R | $MLS_B$ c |
| 16 | 0.125 | 25 | S | 15 | R | 22 | S | 22 | S | 15 | R | 24 | S | M |
| 25 | 0.047 | 29 | S | 16 | R | 22 | S | 24 | S | 30 | S | 26 | S | - |
| 26 | 0.094 | 25 | S | 18 | R | 25 | S | 24 | S | 28 | S | 24 | S | - |
| 28 | 0.064 | 26 | S | 18 | R | 21 | S | 24 | S | 29 | S | 24 | S | - |
| 34 | 0.094 | 24 | S | 16 | R | 20 | S | 21 | S | 25 | S | 22 | S | - |
| 38 | 0.125 | 22 | S | 8 | R | 19 | S | 20 | S | 25 | S | 22 | S | - |
| 55 | 0.064 | 25 | S | 16 | R | 21 | S | 23 | S | 25 | S | 24 | S | - |
| 56 | 0.047 | 22 | S | 15 | R | 19 | S | 20 | S | 21 | S | 22 | S | - |
| 79 | 0.064 | 26 | S | 13 | R | 22 | S | 21 | S | 22 | S | 25 | S | - |
| 85 | 0.064 | 26 | S | 14 | R | 22 | S | 20 | S | 12 | R | 6 | R | $MLS_B$ c |
| 89 | 0.094 | 25 | S | 13 | R | 21 | S | 20 | S | 24 | S | 23 | S | - |
| 95 | 0.094 | 25 | S | 16 | R | 19 | S | 19 | S | 16 | R | 20 | R | $MLS_B$ i |
| 102 | 0.064 | 25 | S | 27 | S | 25 | S | 25 | S | 28 | S | 25 | S | - |
| 120 | 0.094 | 24 | S | 13 | R | 20 | S | 20 | S | 6 | R | 6 | R | $MLS_B$ c |
| 134 | 0.047 | 26 | S | 15 | R | 22 | S | 26 | S | 21 | S | 19 | S | - |
| 135 | 0.064 | 25 | S | 16 | R | 20 | S | 21 | S | 27 | S | 24 | S | - |
| 136 | 0.032 | 27 | S | 14 | R | 23 | S | 25 | S | 18 | I | 27 | S | - |
| 137 | 0.094 | 24 | S | 14 | R | 22 | S | 25 | S | 16 | R | 26 | S | M |
| 139 | 0.064 | 25 | S | 13 | R | 22 | S | 24 | S | 21 | S | 25 | S | - |
| 140 | 0.125 | 25 | S | 16 | R | 22 | S | 24 | S | 12 | R | 25 | S | M |
| 145 | 0.047 | 27 | S | 20 | R | 25 | S | 27 | S | 28 | S | 24 | S | - |
| 151 | 0.064 | 24 | S | 17 | R | 21 | S | 23 | S | 16 | R | 20 | S | M |
| 152 | 0.064 | 25 | S | 13 | R | 22 | S | 21 | S | 6 | R | 6 | R | $MLS_B$ c |
| 155 | 0.047 | 24 | S | 16 | R | 19 | S | 22 | S | 25 | S | 22 | S | - |
| 156 | 0.064 | 25 | S | 12 | R | 21 | S | 23 | S | 27 | S | 24 | S | - |
| 160 | 0.094 | 26 | S | 13 | R | 20 | S | 19 | S | 28 | S | 24 | S | - |
| 167 | 0.047 | 23 | S | 15 | R | 20 | S | 21 | S | 24 | S | 21 | S | - |
| 187 | 0.125 | 23 | S | 12 | R | 19 | S | 20 | S | 6 | R | 6 | R | $MLS_B$ c |
| 15 | 0.094 | 25 | S | 27 | S | 24 | S | 23 | S | 29 | S | 23 | S | - |
| 33 | 0.064 | 25 | S | 14 | R | 20 | S | 21 | S | 6 | R | 6 | R | $MLS_B$ c |
| 39 | 0.064 | 25 | S | 16 | R | 22 | S | 24 | S | 28 | S | 26 | S | - |
| 84 | 0.047 | 27 | S | 14 | R | 23 | S | 20 | S | 10 | R | 6 | R | $MLS_B$ c |
| 144 | 0.094 | 25 | S | 13 | R | 22 | S | 23 | S | 17 | R | 25 | S | M |
| 162 | 0.064 | 26 | S | 13 | R | 19 | S | 23 | S | 6 | R | 6 | R | $MLS_B$ c |

P (1IU) - penicilin; TE (30 µg) - tetracycline; TGC (15 µg) - tigecycline; LEV (5 µg) - levofloxacin; E (15 µg) - erythromycin; DA (2 µg) – clindamycin;$MLS_B$ c - constitutive mechanism of resistance to macrolides, lincosamides and streptogramin B;
$MLS_B$ i - inductive mechanism of resistance to macrolides, lincosamides and streptogramin B; M - M phenotype; (-) - no resistance mechanism;

*Diameter of the inhibition zone [mm] and interpretation of the results of susceptibility testing (accordance with the EUCAST and KORDL recommendations [61, 62]). S - susceptible ; R –resistant.

Supplementary Table S2. Results of serotyping and detection of virulence factors  performed in the assessed strains

| **Serotype** | | **The presence of genes encoding fimbriae** | | | | **The presence of genes encoding surface proteins** | | | | | **Ability to form biofilm** | | |
|---|---|---|---|---|---|---|---|---|---|---|---|---|---|
| **n** | | **Pl-1 +Pl-2a** | **Pl-1 +Pl-2b** | **Pl-2a** | **Pl-2b** | ***epsilon*** | ***rib*** | ***alp2/3*** | ***bca*** | ***alp4***** | **Weak** | **Moderate** | **Strong** |
| **Strains derived from pregnant women (n = 85)** | | | | | | | | | | | | | |
| **Ia** | 15 | 0 | 0 | 15 | 0 | 12 | 3 | 0 | 0 | 0 | 6 | 8 | 1 |
| **Ib** | 7 | 4 | 1 | 1 | 1 | 3 | 1 | 3 | 0 | 0 | 1 | 6 | 0 |
| **II** | 9 | 8 | 1 | 0 | 0 | 0 | 5 | 1 | 3 | 0 | 3 | 6 | 0 |
| **III** | 31 | 14 | 15 | 2 | 0 | 2 | 23 | 6 | 0 | 0 | 14 | 16 | 1 |
| **IV** | 1 | 0 | 1 | 0 | 0 | 1 | 0 | 0 | 0 | 0 | 1 | 0 | 0 |
| **V** | 19 | 18 | 0 | 1 | 0 | 0 | 3 | 16 | 0 | 0 | 9 | 10 | 0 |
| **VI** | 3 | 3 | 0 | 0 | 0 | 0 | 2 | 1 | 0 | 0 | 1 | 2 | 0 |
| **VII** | 0 | 0 | 0 | 0 | 0 | 0 | 0 | 0 | 0 | 0 | 0 | 0 | 0 |
| **Total** | **85** | **47** | **18** | **19** | **1** | **18** | **37** | **27** | **3** | **0** | **35** | **48** | **2** |
| **Strains derived from other adults (n = 44)** | | | | | | | | | | | | | |
| **Ia** | 6 | 1 | 0 | 5 | 0 | 5 | 1 | 0 | 0 | 0 | 5 | 1 | 0 |
| **Ib** | 4 | 4 | 0 | 0 | 0 | 0 | 3 | 0 | 1 | 0 | 2 | 2 | 0 |
| **II** | 6 | 5 | 0 | 0 | 1 | 1 | 2 | 1 | 2 | 0 | 3 | 2 | 1 |
| **III** | 12 | 6 | 4 | 2 | 0 | 0 | 8 | 4 | 0 | 0 | 4 | 6 | 2 |
| **IV** | 1 | 0 | 1 | 0 | 0 | 1 | 0 | 0 | 0 | 0 | 1 | 0 | 0 |
| **V** | 14 | 10 | 2 | 2 | 0 | 0 | 5 | 9 | 0 | 0 | 5 | 8 | 1 |
| **VI** | 0 | 0 | 0 | 0 | 0 | 0 | 0 | 0 | 0 | 0 | 0 | 0 | 0 |
| **VII** | 1 | 0 | 1 | 0 | 0 | 0 | 0 | 1 | 0 | 0 | 1 | 0 | 0 |
| **Total** | **44** | **26** | **8** | **9** | **1** | **7** | **19** | **15** | **3** | **0** | **21** | **19** | **4** |
| **Strains derived from newborn (n = 36)** | | | | | | | | | | | | | |
| **Ia** | 9 | 1 | 0 | 8 | 0 | 9 | 0 | 0 | 0 | 0 | 6 | 3 | 0 |
| **Ib** | 1 | 1 | 0 | 0 | 0 | 0 | 0 | 0 | 1 | 0 | 0 | 1 | 0 |
| **II** | 4 | 3 | 0 | 0 | 1 | 1 | 2 | 0 | 1 | 0 | 2 | 2 | 0 |
| **III** | 11 | 2 | 8 | 0 | 1 | 0 | 9 | 2 | 0 | 0 | 5 | 6 | 0 |
| **IV** | 2 | 1 | 1 | 0 | 0 | 1 | 1 | 0 | 0 | 0 | 1 | 1 | 0 |
| **V** | 9 | 7 | 0 | 2 | 0 | 1 | 4 | 4 | 0 | 0 | 2 | 6 | 1 |
| **VI** | 0 | 0 | 0 | 0 | 0 | 0 | 0 | 0 | 0 | 0 | 0 | 0 | 0 |
| **VII** | 0 | 0 | 0 | 0 | 0 | 0 | 0 | 0 | 0 | 0 | 0 | 0 | 0 |
| **Total** | **36** | **15** | **9** | **10** | **2** | **12** | **16** | **6** | **2** | **0** | **16** | **19** | **1** |

*The *alp4* gene was not found in the tested strains

Supplementary Table S3. Percentage of *Streptococcus agalactiae* strains with different biofilm forming capacity

| Ability to form biofilm* | Isolates [n] | Isolates [%] |
|---|---|---|
| Not a biofilm producer | 0 | 0.0 |
| Weak biofilm producer | 72 | 43.6 |
| Moderate biofilm producer | 86 | 52.1 |
| Strong biofilm producer | 7 | 4.2 |

*The tested strains were classified on the basis of the following criteria: not a biofilm producer: OD ≤ ODc, (all strains with OD values below 0,13); weak biofilm producer ODc: < OD ≤ 2 × ODc, (all strains with OD values above 0,13 and below 0,26); moderate biofilm producer: 2 × ODc < OD ≤ 4 × ODc (all strains with OD values above 0,26 and below 0,52); strong biofilm producer: OD > 4 × ODc (all strains with OD values above 0,52). The mean optical density (OD) of the negative control +3 standard deviations of negative control was considered the cut-off (ODc = 0,13).

Supplementary Table S4. Primer sets used for identification, serotyping and detection of virulence genes in *S. agalactiae*

| | Starter | Primer sequence (5'→3') | Amplicon size (bp) | Annealing temp. (ºC) | Reference |
|---|---|---|---|---|---|
| **Identification** | | | | | |
| GBS cAMP | F | TTTTCACCAGCTGTATTAGAAGTA | 155 | 48.0 | [60] |
| | R | GTTCCCTGAACATTATCTTTGAT | | | |
| **Serotyping** | | | | | |
| Serotyp Ia | F | GGTCAGACTGGATTAATGGTATGC | 521 | 57.0 | [65] |
| *AB028896* | R | GTAGAAATAGCCTATATACGTTGAATGC | | | |
| Serotyp Ib | F | TAAACGAGAATGGAATATCACAAACC | 770 | 57.0 | [65] |
| *AB050723* | R | GAATTAACTTCAATCCCTAAACAATATCG | | | |
| Serotyp II | F | GAGAAGCCCAAGGATGGATT | 188 | 49.2 | DGFM |
| *AY375362* | R | TCCACTATGCTTTTGTAAAATTGG | | | |
| Serotyp III | F | CCACATATGAGAATAAGACTTGC | 187 | 57.0 | [60] |
| *AF163833* | R | CCAACATACAGACCAATAGTATTC | | | |
| Serotyp IV | F | TTTGCTTGTGAGCTACCTGGA | 314 | 52.0 | DGFM |
| *AF355776.1* | R | TCTCCTCCCCAATTTCGTC | | | |
| Serotyp V | F | AACAGAGGCCAATCAGTTGC | 247 | 57.0 | DGFM |
| *AF349539* | R | CTTCCAACGCCACCTCTAAA | | | |
| Serotyp VI | F | GGACTTGAGATGGCAGAAGGT | 523 | 54.0 | DGFM |
| *AF337958.1* | R | CTCGTACAAACTTCGCTTCCAC | | | |
| Serotyp VII | F | GGAGAGAACAATGTCCAGATTACG | 369 | 54.0 | DGFM |
| *AY376403* | R | AGCTGGTCGTGATTTCTACACA | | | |
| **Virulence genes** | | | | | |
| Pl-1 | F | AACCACTAGCAGGCGTTGTCTTTG | 298 | 60.0 | This work |
| *EU929540.1* | R | TGAGCCCGGAAATTCTGATATGCC | | | |
| Pl-2a | F | GCCGTTAGATGTTGTCTTCGTACT | 575 | 60.0 | This work |
| *EU929374.1* | R | TTTACTGCGGTCCCAAGAGCTTC | | | |
| Pl-2b | F | AAGTCTTGACCAAGGATACGACGC | 314 | 60.0 | This work |
| *EU929426.1* | R | ATCGTGTTACTTGCCCTGCGTA | | | |
| Universal | F | TGATACTTCACAGACGAAACAACG | - | 58.0 | [22] |
| Alpha-C | R | TACATGTGGTAGTCCATCTTCACC | 398 | 58.0 | [22] |
| Rib | R | CATACTGAGCTTTTAAATCAGGTGA | 295 | 58.0 | [22] |
| Epsilon | R | CCAGATACATTTTTTACTAAAGCGG | 200 | 58.0 | [22] |
| Alp2/3 | R | CACTCGGATTACTATAATATTTAGCAC | 334 | 58.0 | [22] |

DGFM - primers from a set of primers designed at the Department of Genetics and Pharmaceutical Microbiology.

The primers used in PCR reactions were designed using the Primer3 program (www.http: //bioinfo.ut.ee/primer3-0.4.0/) and checked for the relevant parameters using the Primer-BLAST function (Basic Local Alignment Search Tool) available from the NCBI (National Center for Biotechnology Information). The specificity of the primers was checked in the BLAST Assembled Ref Genomes program https://blast.ncbi.nlm.nih.gov/Blast.cgi.

To identify serotypes II, IV, VI and VII, single PCR reactions were carried out, whereas PCR multiplexes were optimized for serotypes Ia, Ib, III and V.Genes encoding various types of fimbriae (Pl-1, Pl-2a, Pl-2b) were detected using single standard PCR reactions.In contrast, the identification of genes encoding surface proteins from the Alp family was carried out in the multiplex PCR reaction using a modified methodology described by Creti R et al. [22]. Also to identify genes encoding fibrinogen-binding proteins, a multiplex PCR reaction was used.
